# Cerebrotendinous xanthomatosis (a rare lipid storage disorder): a case report

**DOI:** 10.1186/s13256-016-0882-y

**Published:** 2016-04-19

**Authors:** Syed Mohd Razi, Abhinav Kumar Gupta, Deepak Chand Gupta, Manish Gutch, Keshav Kumar Gupta, Syeda Iqra Usman

**Affiliations:** 1 Department of Endocrinology, Lala Lajpat Rai Memorial Medical College, Garh Raod, Meerut, 250004 Uttar Pradesh India; 2 Jawaharlal Nehru Medical College, Aligarh Muslim University, Aligarh, 202002 Uttar Pradesh India

**Keywords:** Cerebrotendinous xanthomatosis, *CYP27A1*, Xanthoma, Chenodeoxycholic acid, Cholestanol

## Abstract

**Background:**

Cerebrotendinous xanthomatosis is a very rare autosomal recessive lipid storage disorder affecting bile acid biosynthesis. It is manifested by subtle neurological and non-neurological symptoms due to abnormal tissue lipid deposition. Diagnosis is usually delayed but early diagnosis and replacement therapy can prevent devastating neurological sequelae.

**Case presentation:**

We present a case of a 25-year-old Asian Indian woman who presented with gait difficulty, fusiform swellings of bilateral tendo-Achilles and infrapatellar tendons, along with history of bilateral cataract surgery 1 year earlier. The diagnosis was made on the basis of clinical, biochemical, imaging, and histopathological analysis and replacement therapy was started.

**Conclusions:**

The peculiarity of the present case is the absence of any neurological manifestations which are usually the early clues to the diagnosis of cerebrotendinous xanthomatosis. The present case report emphasizes the fact that early age bilateral cataracts along with bilateral tendo-Achilles xanthomas can be early pointers toward the diagnosis of cerebrotendinous xanthomatosis.

## Background

Cerebrotendinous xanthomatosis (CTX) is a rare autosomal recessive lipid disorder: phenotype Online Mendelian Inheritance in Man (OMIM)#213700, gene OMIM#606530 [1]. It is caused by mutations in gene *CYP27A1* located on the chromosome 2q33-qter causing lack of enzyme mitochondrial sterol 27-hydroxylase [1]. *CYP27A1* plays a pivotal role in cholesterol side chain oxidation during the synthesis of chenodeoxycholic acid (CDCA) which is a bile acid [2]. Therefore perturbations in *CYP27A1* gene result in reduced enzymatic activity causing impairment of cholesterol side chain oxidation finally culminating in excessive production and abnormal deposition of cholestanol in various tissues [1, 2]. The classical triad of the syndrome consists of premature bilateral cataract, tendon xanthomas (predominantly involving tendo-Achilles) and various neurological abnormalities [3]. A recent study by Appadurai *et al*. [2] stated that in the absence of epidemiological studies the disease is considered to be exceedingly rare but it is actually underreported. This group studied the Exome Aggregation Consortium (ExAC) cohort of 60,000 unrelated adults globally for the frequency of 57 reported pathogenic variants of *CYP27A1* along with 29 additional variants through bioinformatics; they estimated that the incidence of CTX ranged from 1:134,970 to 1:461,358 in Europe, 1:263,222 to 1:468,624 in Africa, 1:71,677 to 1:148,914 in the USA, 1:64,267 to 1:64,712 in East Asia, and 1:36,072 to 1:468,624 in South Asia [2]. However, another recent study by Ragno *et al*. stated that more than 300 cases have been reported so far with approximately 50 mutations with little correlation between genotype and phenotype [4]. The mean age of onset of the symptoms is 19 years while the mean age at diagnosis is 35 years representing a lag of 16 years in the diagnosis [5]. A recent study of 19 patients from 15 unrelated Italian families by Mignarri *et al*. estimated the median age at diagnosis to be 32 years while the mean age was 32.5±10.4 years [6]. Replacement therapy with CDCA delays or even reverses the progression of the disease [3]; however, because there is a lag period between the onset of symptoms and the mean age of diagnosis [1], early diagnosis is required to prevent devastating neurological sequelae and other complications such as premature atherosclerosis and osteoporosis. So a high index of suspicion should be kept in any patient encountered with the classical triad as illustrated in the present case report.

## Case presentation

A 25-year-old Asian Indian woman presented with complaints of swellings behind both her ankles and in front of both her knees for the last 2 years. These swellings had an insidious onset; they were painless, gradually progressive, and caused difficulty in walking for the last 1 year. She had history of bilateral cataract surgery 1 year earlier. There was no history of childhood diarrhea, seizures, cerebellar symptoms, psychiatric manifestations, mental retardation, or premature atherosclerosis. There was no family history of similar complaints.

**Fig. 1:**
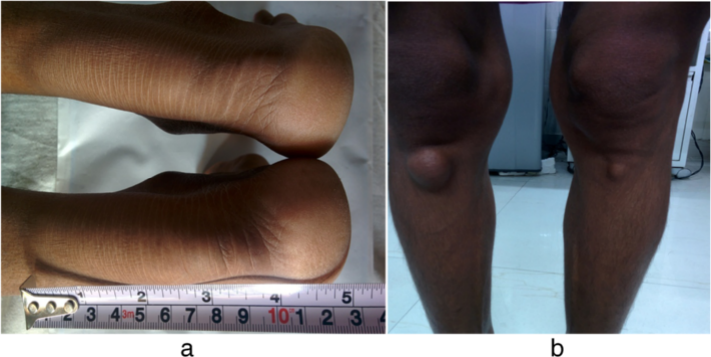
**a** Bilateral fusiform tendo-Achilles xanthomas: right 10×4.5 cm, left 8×3 cm. **b** Bilateral infrapatellar tendon xanthomas: right 2×1.5 cm, left 1.5×1.2 cm

On examination, she had firm, non-tender, fusiform swellings over bilateral tendo-Achilles (right 10×4.5 cm, left 8×3 cm) and bilateral infrapatellar tendons (right 2×1.5 cm, left 1.5×1.2 cm) as shown in Fig. 1a, b. Her general examination was unremarkable except for bilateral pseudophakic eyes. On neurological examination, her higher mental functions were normal. Her pupils were normal sized and normally reactive. Her sensory system was unremarkable. Her muscle bulk, tone, power, coordination, and other motor system functions were normal. All her cranial nerves were normal. Her cardiac, abdominal and respiratory examinations were also unremarkable.

**Fig. 2:**
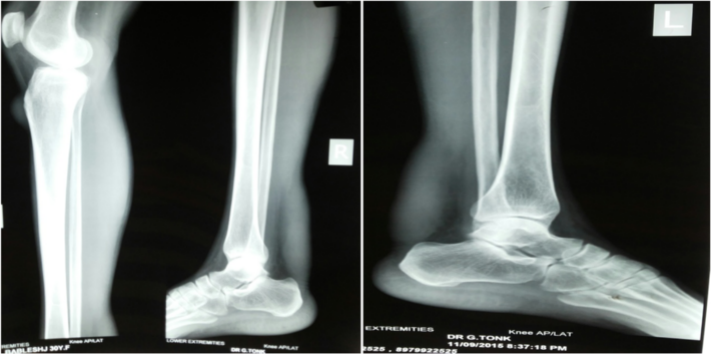
X-ray showing soft tissue thickening of bilateral tendo-Achilles

**Fig. 3:**
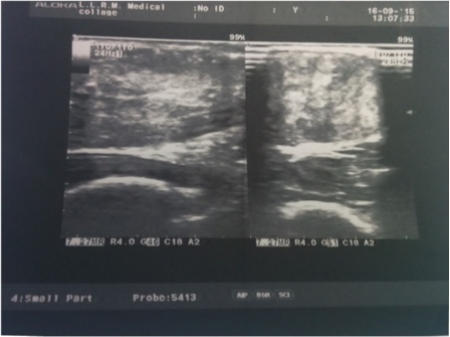
Ultrasonography of tendo-Achilles showing anteroposterior thickness of 7.5 mm with loss of normal tendon appearance and multiple hypoechoic foci within tendon

**Fig. 4:**
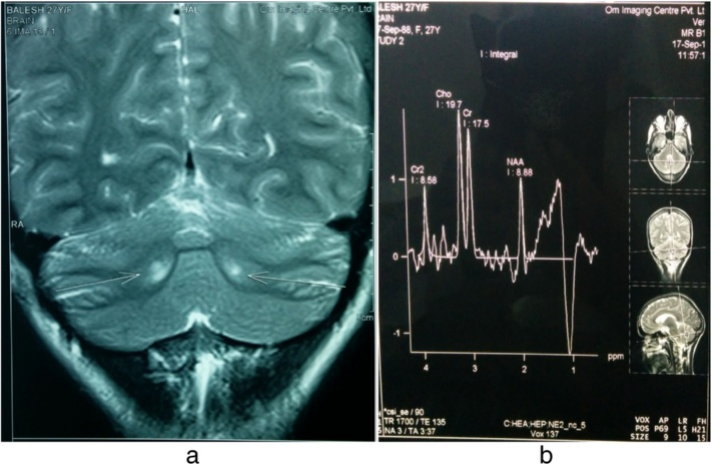
**a** Brain magnetic resonance imaging T2 sequence showing hyperintensities in bilateral dentate nuclei. **b** Magnetic resonance spectroscopy showing high choline and low N-acetylaspartate/creatine peaks

**Fig. 5:**
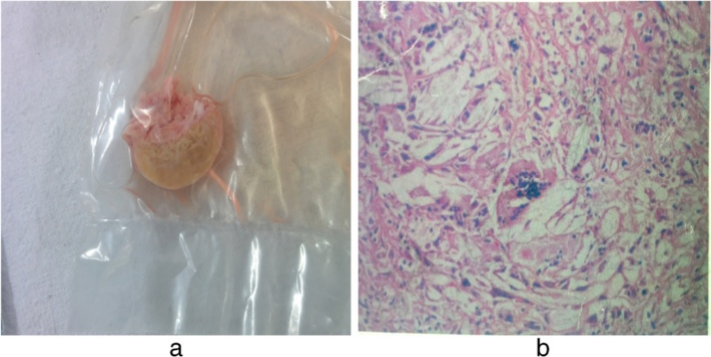
**a** Gross – excisional biopsy specimen from right infrapatellar tendon xanthomas measuring 2×2×1 cm with gray, brown, soft tissue. **b** Microscopic analysis – showing foamy cells admixed with inflammatory cells and giant cells surrounding cholesterol clefts

Her hemogram, renal function test, hepatic function test, serum electrolytes, and fasting lipid profile were within the normal limits. Her serum cholestanol level was 4.27 mg/dl (normal value 0.02 to 0.12 mg/dl). Serum and urinary bile alcohol could not be done because of unavailability. Electrocardiography, two-dimensional echocardiography, spirometry, and nerve conduction study were unremarkable. X-rays of both her legs revealed soft tissue thickening in bilateral ankles posteriorly and overlying right tibial tuberosity as shown in Fig. 2. Ultrasonography of bilateral tendo-Achilles revealed anteroposterior thickness of 7.5 mm and loss of normal tendon appearance with multiple hypoechoic foci within the tendon as shown in Fig. 3. Magnetic resonance imaging (MRI) of her brain revealed T2 and fluid-attenuated inversion recovery (FLAIR) hyperintense signals in the region of the dentate nucleus of both her cerebellar hemispheres with high choline and low N-acetylaspartate (NAA)/creatine peaks on magnetic resonance spectroscopy (MRS) as shown in Fig. 4a, b. She underwent excisional biopsy of her right infrapatellar which yielded a gray, brown, soft tissue measuring 2×2×1 cm on gross examination as shown in Fig. 5a. Microscopic examination revealed foamy cells admixed with inflammatory cells and giant cells surrounding cholesterol clefts as shown in Fig. 5b. On the basis of these findings the diagnosis of CTX was made and she was started on replacement with CDCA 250 mg three times a day, ursodeoxycholic acid 300 mg three times a day, and atorvastatin 10 mg at bedtime.

## Discussion

CTX is a rare autosomal recessive inborn error of bile acid synthesis first reported by Von Bogaert in 1937. The disease is caused by mutation in gene *CYP27A1* located on chromosome 2q33-qter leading to a lack of mitochondrial sterol 27-hydroxylase enzyme at the inner mitochondrial membrane in almost all cells of the body [7, 8]. Absence of sterol 27-hydroxylase enzyme activity causes deficiency of CDCA and cholic acid leading to a loss of feedback inhibition of rate-limiting enzyme cholesterol 7α-hydroxylase (CYP7A1) of bile synthesis, which results in excess production of cholestanol which gets deposited in various tissues [1–3].

The diagnosis is delayed due to subtle manifestations and the rarity of the disease [1]. The disease has multiorgan involvement such as bilateral premature cataracts, intractable childhood diarrhea, tendon xanthomas, neurological abnormalities, and premature atherosclerosis. The usual manifestations are due to deposition of excess cholestanol and cholesterol in the affected tissues [1, 3]. Our patient presented with bilateral tendo-Achilles tuberous xanthomas along with premature bilateral cataracts but no neurological abnormalities.

Familial hypercholesterolemia and sitosterolemia are the two important differential diagnoses of CTX. CTX is differentiated clinically from other disorders of lipid metabolism because of juvenile cataracts, progressive neurological symptoms, mild pulmonary insufficiency, and increased levels of cholestanol [9]. In the absence of tendinous xanthomas, CTX may be confused with Marinesco–Sjögren syndrome as it also presents with early age cataracts, cerebellar ataxia, neuromuscular weakness, and mental retardation but it can be differentiated from CTX by short stature, skeletal anomalies and hypergonadotropic hypogonadism [10, 11].

The laboratory findings include a normal or reduced serum cholesterol level, increased serum cholestanol, increased 7α-hydroxy-4-cholesten-3-one (7αC4), increased lathosterol, increased plant sterols (campesterol, sitosterol), increased serum and urinary bile acid alcohols but reduced serum CDCA and 27-hydroxycholesterol (27-OHC) [6]. Typical MRI findings are bilateral T2 and FLAIR hyperintense, nonhomogeneous signals in dentate nuclei and surrounding cerebellum. There are diffuse or focal white matter abnormalities along with cerebral and cerebellar atrophy [1, 3, 10]. MRS studies reveal increased lactate and lipid peaks in FLAIR sequence-hypointense lesions, and diffusely decreased NAA peaks. Microscopy reveals foamy cells admixed with inflammatory cells and giant cells surrounding cholesterol clefts [1]. These laboratory findings exactly correlate with those of our patient in the present discussion.

The treatment consists of replacement therapy, surgery and other symptomatic therapies. The replacement therapy can prevent or even reverse neurological complications and involves administration of bile acids such as CDCA, ursodeoxycholic acid, cholic acid, and taurocholic acid. Among these, CDCA is considered the drug of choice. Hydroxymethylglutaryl-CoA (HMG-CoA) synthase inhibitors are considered to enhance the effect of replacement therapy. Surgery may deteriorate gait imbalance and does not prevent neurological complications [1].

## Conclusions

CTX is a rare and underreported lipid storage disorder. Although it is medically manageable, a delay in diagnosis can be devastating for the patient so early diagnosis based on a high index of suspicion is imperative for better management.

## Consent

Written informed consent was obtained from the patient for publication of this case report and any accompanying images. A copy of the written consent is available for review by the Editor-in-Chief of this journal.
